# Is Raising Your Grandchild Bad for Your Health? The Association Between Custodial Grandparent Status and Health Biomarkers in Taiwanese Grandparents

**DOI:** 10.3390/ijerph17051753

**Published:** 2020-03-07

**Authors:** Zoe N. Fokakis, Danielle K. Nadorff, Ian T. McKay

**Affiliations:** Department of Psychology, Mississippi State University, P.O. Box 6161, Mississippi State, MS 39762, USA; znf4@msstate.edu (Z.N.F.); itm24@msstate.edu (I.T.M.)

**Keywords:** custodial grandparents, biomarkers, health

## Abstract

Data from two waves of the Social Environment and Biomarkers of Aging Study in Taiwan were analyzed to determine the effects of custodial grandparenting on health in a longitudinal sample. Self-reported measures on respondents’ perception of their health, six health biomarkers, the presence of twelve diseases, and a measure of stress were included. Custodial Grandparents (CGPs) were significantly more likely to report worse health than their peers. However, there were no significant differences in biomarkers, and CGPs were only significantly different from non-custodial grandparents (nCGPs) regarding lower respiratory disease. Results suggest that CGPs do not have significantly worse health than nCGPs, but report feeling less healthy. This disparity is suspected to be due to energy levels or stress sources not assessed by the variables in the original study. These results and their implications based upon the stress-coping model elucidate the need to design interventions that incorporate the East Asian cultural values and practices in order to promote better health outcomes for CGP populations overall.

## 1. Introduction

Approximately 10% of all grandparents living in the United States live with their grandchildren. Of these 6.7 million grandparents, more than 4.9 million were in households that were headed by the grandparents themselves, and about one third of co-resident households had no middle generation present [1]. The US Census Data in 2010 also found that grandparents living with a grandchild were typically younger and less educated and were more likely to be in poverty than those grandparents who did not live with a grandchild [1]. As life expectancy continues to increase, the population of co- resident and custodial grandparents has rapidly increased as well. From 2000 to 2012 the number of co-resident grandparents rose from 5.8 million to 6.7 million, and the number of grandparent caregivers rose from 2.4 million to 2.7 million [2]. Taking on the responsibility of caring for one’s grandchild can place an added, unexpected strain on older adults. This strain can manifest in physical, psychological, and emotional health complications or in their financial situation.

Dr. Meredith Minkler and Dr. Esme Fuller-Thomson found that custodial grandparents (CGPs) were significantly more likely to have lower self-rated satisfaction with their health than non- custodial grandparents (nCGPs). CGPs also were 50% more likely to have limitations in at least one of five tested activities of daily living [3]. However, a number of studies conducted in Asia have reported conflicting results. A longitudinal study across Taiwan found that long-term multigenerational caregivers (caregiving grandparents whose adult children live in the same household) were more likely to report better self-rated health [4]. Another longitudinal study in China found that both previous CGPs and repeated CGPs had better self-rated health than their noncustodial peers [5]. This relationship was also mediated by emotional support received, which may help to explain the disparity between Eastern CGP populations and those from Western cultures.

CGPs also appear to experience increased stress and family strain. In a sample of 485 Ohioan grandmothers, Musil and colleagues found that custodial grandmothers reported more stress, intrafamily strain, and perceived family functioning problems over a two-year period than non- custodial grandmothers [6]. In another study conducted by Musil, custodial grandmothers reported higher levels of parenting stress and lower levels of social support than non-caregiving grandmothers [7]. This stress may be due in part to low self-confidence and/or poor perceptions of their parenting abilities when compared to their noncustodial peers.

Previous research has also shown that CGPs tend to have higher levels of depressive symptoms. One study of a national sample (n = 977) found that grandparents experienced an increase in depressive symptoms when a grandchild was in residence compared to when they did not have a grandchild in residence [8]. However, this is another finding with conflicting results from a number of Asian studies. For example, in a longitudinal study of Korean grandparents, those who began caregiving or were caregivers for the full three-year period observed reported reduced depressive symptoms compared to nCGPs [9].

Whether these factors influenced Ku’s results or not, it is generally agreed upon that CGPs do in fact experience greater life satisfaction than nCGPs. One survey of grandparent needs, wellbeing, and health found that CGPs derived satisfaction in three aspects of their role: “child-centered nurturance of an at-risk grandchild, dyadic engagement in shared activities with their grandchild, and the personal grandparent-centered pleasure benefits to be had from fulfilling a grandcarer role” [10]. This effect was even stronger in formal CGPs who had legal custody of the grandchildren than it was for informal CGPs.

**Figure 1:**
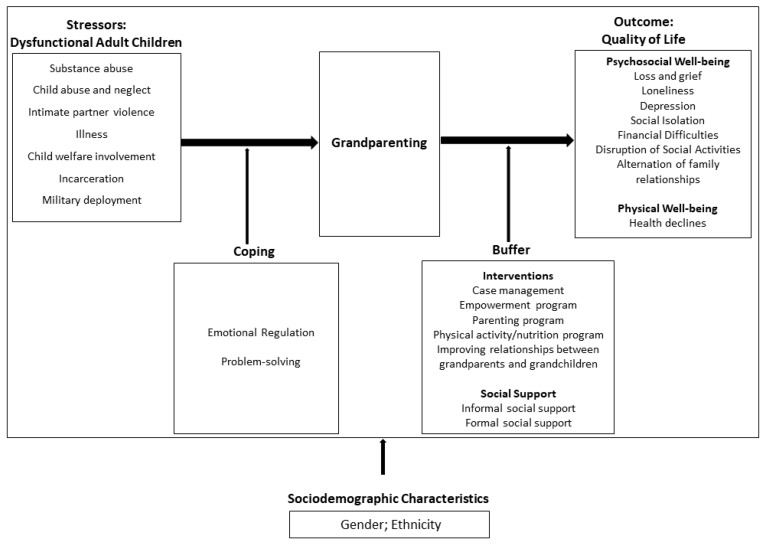
Choi, Sprang, and Eslinger’s stress-coping model.

Choi and colleagues’ stress-coping model (Figure 1) depicts the many factors that influence both the process of becoming a CGP as well as the outcomes of this new life role, both in terms of physical and psychosocial well-being. As such, the health of this study’s population will be viewed in light of the variables presented by the stress-coping model, including stress appraisal, formal and informal social supports, and cultural factors [11].

All of these variables illustrate why there are such differing results when we compare CGPs’ health and their perceived health. Additional confounds may also be influencing this relation. For example, thus far, an exhaustive literature seach has revealed no studies on CGPs’ health that have included the use of biomarkers as a measurement of health. While perceived health and the ability to complete activities of daily living without limitation is important, it often does not get to the root of health issues, and therefore these results may be misleading. Self-rated health especially presents stark inaccuracies.

In their review, Dr. Grinstead and colleagues found that many caregivers would report good or even excellent health, then mention a severe chronic illness in their interviews [12,13]. A recent qualitative study also found that the majority of CGPs interviewed had at least one serious chronic health complaint which caused increased fatigue and stress when tasked with raising their grandchildren. The respondents state, “It’s [their] age and physical capacity that affects [them] the most,” and that custodial grandparenting “sucks everything out of you,” [12]. While this study begins to touch on the realities of CGP health, there is still a need for a quantitative analysis of health biomarkers in order to give a more accurate picture of these grandparents’ current health and their health trajectories. In this study, we aim to begin to fill this deficit, by using known biomarkers of health and the presence of fourteen diseases and disorders to compare CGPs’ health to the health of nCGPs. The biomarkers examined in this study included cortisol levels, IL-6 protein levels, triglycerides, cholesterol, and leukocyte telomere length.

Cortisol is known as the primary stress hormone because it inhibits bodily functions that could interfere with a person’s fight-or-flight response. Cortisol levels are affected by the adrenal glands during times of stress and hormonal changes. Specifically, cortisol increases blood glucose levels, alters the immune response, and suppresses the digestive system, reproductive system, and growth processes. These conditions are necessary for short-term stress responses but are damaging when maintained for a long period of time, as in the case of chronic stress [14]. A new study has also shown that high cortisol levels may be associated with cognitive and physical decline in patients with late- life depression [15].

Interleukin 6 (IL-6) is a protein with many different functions; it acts as both a pro-inflammatory cytokine and an anti-inflammatory myokine. IL-6 can activate differentiation of B cells into antibody- producing cells, affect protein synthesis on hepatocytes, enhance growth of fusion cells between plasma cells and myeloma cells, and exhibit antiviral activity. IL-6 is produced and released by muscle cells as a necessary response to infections and tissue injuries; however, unregulated synthesis of IL-6 plays a pathological role in chronic inflammation and autoimmunity. The many functions of IL-6 mean that its unregulated production can lead to hypoferremia, anemia, osteoporosis, and eventually the progressive deterioration of various organs [16].

Cholesterol and triglyceride levels are two commonly referenced biomarkers when examining one’s health. While, both types of lipids are necessary for proper function, excessive amounts of either can lead to significant cardiovascular complications. Triglycerides are stored in fat cells and released for energy between meals. If a person consistently consumes more calories than they are metabolizing, triglycerides may build up in fat cells or continue to circulate through the blood. This can lead to arteriosclerosis and pancreatitis. High triglycerides can also be a warning sign of stroke, heart disease, heart attack risk, and metabolic syndrome [17]. Cholesterol operates in a much different manner but can lead to many of the same health risks. Cholesterol is used to maintain cell structure and is transported through the body in the blood. High cholesterol can leave fatty deposits in the blood vessels, which can lead to atherosclerosis, angina, cardiovascular disease, heart attack, or a stroke [18].

Telomeres are repeating sequences of nucleotides (5’-TTAGGG-3’) on the ends of DNA that prevent the loss of genetic material during replication. Telomeres were first discovered in 1978 and have since been studied as indicators of current health and predictors of future health. In a 2013 study by Sanders and Newman, telomere length was determined to be able to predict the rate of aging by reflecting levels of cellular senescence and oxidative stress. This is because oxidative stress causes telomeres to shorten, possibly due to a temporary stalling of the replication machinery. As telomeres become critically short, cells become senescent. A large accumulation of senescent cells will lead to inflammation and subsequently chronic illnesses such as Chronic Obstructive Pulmonary Disease (COPD), diabetes, and tumor development [19].

Though it is unknown whether one’s health influences telomere length or not, telomeres have been shown to be influenced by a number of lifestyle factors. Shorter telomeres have been associated with higher anticipatory threat appraisals, lifetime depression exposure, and low social support in old age. A meta-analysis conducted by Schutte and Malouff found that higher levels of perceived stress were associated with shorter telomeres (*p* < 0.001) [20]. Another study found that higher anticipatory threat appraisals were associated with shorter telomere length (*p* = 0.03), but challenger appraisals and retrospective threat appraisals showed no association with telomere length [21]. Yet another study found that individuals with major depressive disorder (MDD) had significantly reduced leukocyte telomere length (LTL) compared to those without it. This difference was maintained by both treatment resistant and responsive MDD patients, and there was no correlation with antidepressant treatment and LTL [22]. It has even been suggested that telomeres may be “a ‘missing link’ for understanding morbidity and mortality in depression” [23]. One study found that for adults aged 65 and older, low social support was associated with significantly shorter telomeres, even with all covariates adjusted for [24]. Uchino and colleagues also found that a higher number of ambivalent relationships in a person’s social network was related to shorter telomere length, regardless of the amount of positive or negative relationships a respondent had [25].

### 1.1. Current Study

This study will add to existing research by using biomarkers, as well as the presence of certain diseases, as indicators of health, which have not yet been examined in the custodial grandparent population. The overall purpose of this project is to add to the existing knowledge and current understanding that we have of custodial grandparenting and the pros and cons that are associated with this role. Further, custodial grandparents are a growing demographic, and as we learn more about their strengths, needs, and the challenges they face, we will be better able to advocate for institutional programs to suit their needs.

### 1.2. Hypotheses

**Hypothesis** **1.** *Telomeres of custodial grandparents will be shorter than those of their non-custodial peers*.

**Hypothesis** **2.** *CGPs will exhibit significantly more diseases and disorders than nCGPs. CGPs are also expected to have significantly different biomarker levels from their nCGP peers*.

## 2. Design and Methods

### 2.1. Participants

Data were taken from Waves One (2000) and Two (2006) of the Social Environment and Biomarkers of Aging Study (SEBAS) in Taiwan, which is an extension of the Taiwan Longitudinal Study of Aging [26]. Wave One included participants aged 54 and older in 2000. Wave Two contained a refresher cohort of participants aged 53–60 in 2006, in addition to the original respondents.

Respondents were categorized for this study as either Custodial Grandparent (CGP) or non- Custodial Grandparent (nCGP). CGPs were those participants who reported living with grandchildren but not living with married children in either Wave One (*N* = 53) or Wave Two (*N* = 54) or as CGP across either wave (*N* = 97, *M* = 68.62 years, *SD* = 8.98 years). nCGPs were all other participants who reported having a grandchild, but either did not report living with grandchildren or reported living with grandchildren and married children (Wave One: *N* = 465; Wave Two: *N* = 799, Overall nCGP: *N* = 956, *M* = 64.49 years, *SD* = 9.598).

### 2.2. Measures

Measures included Self-Reported Health (composed of a total score from three self-reported questions assessing current state of health, health compared to a year ago, and health compared to people your age) and twelve questions regarding a diagnosis of a particular disease or disorder. Five health biomarkers were also examined, the data for which were originally obtained by blood and urine analysis. The measure of self-reported health included the questions “Regarding your current state of health, do you feel it is excellent, good, average, not so good, or poor?”, “Compared to this time last year, is your health better, about the same, or worse?”, and “Compared to most people your age, do you feel your health is better, about the same, or worse?”. These answers were summed for a Wave 1 participant mean of 7.18, *SD* = 1.74. Chronbach’s alpha = 0.623, and a Wave 2 participant mean of 7.06, *SD* = 1.79, Chronbach’s alpha = 0.632. These alphas are considered low but within acceptable range [27]. Lower numbers on the scale equal better self-estimated health values.

The diseases and disorders assessed included the following: high blood pressure, diabetes, heart disease, stroke, cancer or malignant tumor, lower respiratory disease, arthritis and rheumatism, ulcers or stomach ailment, liver or gall bladder disease, kidney disease, gout, and spinal spurs. The answers for each item under the diseases and disorders were dichotomous, “yes” or “no.” Biomarkers examined included cortisol levels, IL-6 protein analysis, total triglycerides, cholesterol, and leukocyte telomere length. The urinary cortisol levels and IL-6 protein concentration had previously been recoded so that the baseline assay sensitivity was coded at –999. Leukocyte telomere length (LTL) was recorded as the T/S ratio. An LTL_Flag variable was also included to mark any possibly unreliable LTL values, including a missing sample, sample not sent to University of Washington for measurement, failed PCR, or if the sample was outside of the range from 1 ng to 6 ng.

Participants were asked multiple questions about their stress concerning different areas of their lives. These included stress about respondents’ own health, own finances, own job, family relations, family’s health, family’s finances, family’s work, and family’s marital situation. For each item, respondents were asked if they felt stress about the situation, how much stress they felt about it (rated as 1: “some stress” or 2: “a lot of stress”), the number of years they had been stressed about this topic, and the number of months they had felt stressed about this topic. These items were combined to create an Overall Stress variable for Waves One and Two.

### 2.3. Analytical Methods

For the analysis of leukocyte telomere length (LTL), only Wave One was observed, due to the absence of this data from Wave Two. Thus, for this variable CGPs were defined as those participants who responded as living with grandchildren but not living with married children in only Wave One. Additionally, the dataset was filtered of any respondents whose LTL measurement was deemed to be possibly unreliable (*CGPs N* = 51; *nCGPs N* = 423). A one-way between subjects ANOVA was performed for this variable. For all other biomarker variables, a repeated measures ANOVA was conducted using the variable CGPs across Either Wave. A logistic regression was run for each item assessing the presence of disease and disorders. To assess stress, each question regarding how much stress the respondent experienced concerning each item was coded as one of the following: 0–“no stress,” 1–“some stress,” and 2–“a lot of stress.” Missing values were coded as 0 if the respondent answered related questions on that stress item or were coded as missing if that item was not responded to at all. For example, if a respondent answered ‘no’ to the question “Do you feel stress about your family’s health?” and therefore had a missing value for the question “How much stress do you feel about your family’s health?,” then the missing value would be coded as a 0 to indicate that no stress was felt regarding that item. All eight stress items were combined into an “Overall Stress” variable, which was formed by the summation of the individual items. This variable could therefore exhibit values ranging from 0 to 16. This procedure was done for both Wave 1 and Wave 2 to create two Overall Stress variables. Bivariate correlations were then run for both Overall Stress variables against all of the disease and disorder items, as well as the five biomarker items.

## 3. Results

A repeated measured ANOVA found that self-rated health decreased over time, *F*(1, 486) = 4.79, *p* < 0.05, partial η^2^ = 0.01. The values of self-rated health from Wave One (*M* = 7.06, *SD* = 0.11) to Wave Two (*M* = 7.34, *SD* = 0.12) showed a statistically significant mean difference of –0.283, 95% CI [–0.53, 0.03], *p* < 0.05. As higher values within this scale indicate poorer health, this means that participants’ self- rated health worsened over time. However, the interaction of custodial status by time was not significant. To follow up, one-way ANOVAs were conducted on each wave’s self-rated health using custodial status at that timepoint. For Wave One, there was a significant effect of custodial status on self-rated health [*F* (1, 509) = 4.64, *p* < 0.05]. There was not a significant effect of custodial status on self- rated health at Wave Two.

Binary logistic regressions were run on the 12 diseases and disorders reported. The logistic regression model for the dependent variable of Lower Respiratory Disease was statistically significant, χ^2^(4) = 19.39, *p* < 0.001, explained 4.5% of variance, and correctly classified 90.2% of cases. Controlling for age, sex, and SES, CGPs were 1.95 times more likely than nCGPs to have been diagnosed with lower respiratory disease. Contrary to our original hypothesis, none of the remaining 11 diseases or disorders differed based on caregiving status. A repeated measures ANOVA was run for four of the five biomarkers (cortisol, IL-6, triglycerides, and cholesterol), and a one-way between subjects ANOVA was conducted for leukocyte telomere length. None of these biomarkers exhibited a significant difference between groups. These results did not support either of our hypotheses, but rather indicated that while CGPs perceived themselves as having worse health than their peers, their actual health was not significantly different from nCGPs in any respect except for the probability of having lower respiratory disease.

In an attempt to account for the difference in perceived health, the Overall Stress reported in Wave 1 and Wave 2 was examined for correlations to any of the health indicators. It was found that Overall Stress Wave 1 was positively correlated with LTL (*R ^2^=* 0.076) and Triglycerides Wave 1 (*R^2^*= 0.069), as well as being correlated to 5 of the 12 diagnoses in Wave 1 and 10 diagnoses in Wave 2. Overall Stress Wave 2 was not correlated to any of the biomarkers but was positively correlated with Lower Respiratory Disease Wave 1 (*R^2^*= 0.072), Arthritis or Rheumatism Wave 1 (*R^2^*= 0.086), Liver or Gall Bladder Disease Wave 1 (*R^2^*= 0.080), and Spinal Spurs Wave 1 (*R^2^*= 0.092). These results indicated a complex interaction between health status and stress levels, which can be seen in Table 1 and Table 2. However, a repeated measures ANOVA found no significant interaction between custodial status and stress levels (*p* = 0.061).

## 4. Discussion and Implications

Within our sample, CGPs rated their current health as significantly worse than their peers at Wave One, a finding contrary to the results of Chen and Liu [28]. However, there was little to no actual difference in their health compared to their nCGP counterparts, with the only significant group difference being for lower respiratory disease. It is unclear as to why CGPs were significantly more likely to report having lower respiratory disease. Most causes of chronic lower respiratory disease (smoking, exposure to dust and fumes) are associated with lower socioeconomic status and increased age; however, the differences were still present after controlling for these factors. While stress levels were examined as a possible explanation for these results, no relationship between Overall Stress and custodial status was observed. It is interesting to note that our findings differ from previous studies that reported no deficit in self-reported health among custodial grandmothers [7]. It is likely that this disparity may be due to cultural differences present within the two samples, as previous research has found that cultural norms may play a role in caregiver health [28]. Further, values present in eastern cultures (e.g., collectivism, filial piety, interdependence, and myth of illness) differ drastically from cultural norms in more westernized societies (e.g., independence, individualism, self-criticism). These differences may be contributing to differences in the perceptions of caregiving and its impact on physical health.

### 4.1. Translational Significance

In light of Choi, Sprang, and Eslinger’s stress-coping model of custodial grandparenting outcomes (Figure 1), our results suggest that ethnicity not only influences the process of becoming a CGP, but also in mediating the outcomes of this life role [11]. Choi and colleagues’ model depicts ethnicity as being a factor which informs the likelihood of an individual becoming a CGP as well as how the individual responds to the stressors that lead to grandparenting. The increasingly stark contrast of health outcomes in Western and East Asian populations demonstrates that cultural values may be playing a larger role in the outcomes of CGPs. The results found in this study suggest that cultural values such as high vertical collectivism and increased social participation may act as additional buffers to alleviate the stress of the new caregiver role. The importance of these values in Taiwanese culture and their overall impact on health outcomes are demonstrated in Lee and colleagues’ analysis of active engagement in Taiwanese older adults, which found that both men and women who were active in social groups demonstrated better physical health, and that involvement in social groups was predictive of future physical health for women [29]. These results and their implications based upon the stress-coping model elucidate the need to design interventions that incorporate the East Asian cultural values and practices in order to promote better health outcomes for CGP populations overall. Factors such as these are recommended for future study.

### 4.2. Limitations

Generalizability across cultures is limited, as the population comes entirely from Taiwan. In Taiwan it is quite common for multiple generations of a family to live within the same household, and for grandparents to have a significant role in raising their grandchildren. This could be a potential reason that differences were not seen in the health outcomes of CGPs and nCGPs. Though it may seem problematic that our study utilized a more westernized cultural context of grandparent caregiving within a predominately Asian sample, there has been a paucity of grandparent caregiver research that has examined biomarkers and the presence of certain diseases, as indicators of health. Thus, the exploratory nature of the study should be considered when attempting to generalize findings to other populations. Additionally, the available method of coding for CGPs was a limiting factor for this study. While the survey questions were helpful in narrowing down who could be a CGP, there was no explicit question assessing whether or not one had primary caregiving responsibilities for a grandchild. The current study defined CGPs as those living with grandchildren, but not with married children. This leaves room for error, as in some cases the grandchildren, whose ages are not given, may be caring for their grandparents or the middle generation may still be present in the household as an unmarried child of the respondent. The length of time that the respondent had been living with their grandchildren was also not addressed by the survey. Furthermore, when examining how stress influenced any health outcomes, the measures used were not defined by any particular stress scale. The question of “some stress” versus “a lot of stress” is subjective and not as precise as using a Likert scale for a validated stress measure, such as the Perceived Stress Scale (PSS).

### 4.3. Future Directions

It is recommended that future studies assess the relation between grandparents’ caregiving status and physical health using a diverse nationwide sample and longitudinal design. Due to the exploratory nature of the study, future studies utilizing a diverse sample will provide more generalizable results, especially with regards to Western cultures. A diverse sample would also help to elucidate how findings can best inform practice, research, or policy across cultures. Future research should also employ a longitudinal design. If utilized, it would be beneficial to include the same measures throughout the course of the study. This would allow a two-way analysis of any biomarkers or other factors used to predict health outcomes. Additionally, it could be beneficial to include the PSS as well as the Geriatric Depression Scale to determine if there is any covariance of stress and depression with physical health in the CGP population.

## Figures and Tables

**Table 1:** Correlations between stress at both waves and diseases/biomarkers at Wave 1.

	Stress W1	Stress W2
Arthritis/Rheumatism W1	0.076 *	0.086 *
Cancer/Malignant Tumor W1	−0.009	−0.399
Diabetes W1	−0.023	0.043
Gout W1	0.050	0.036
Heart Disease W1	−0.081	−0.063
High Blood Pressure W1	0.124 *	0.132 *
IL-6 EIA W1	0.081 **	0.074 *
Kidney Disease W1	0.100	0.157
Liver/Gall Bladder W1	0.087 **	0.080 *
Lower Respiratory Disease W1	0.102 **	0.072 *
LTL	0.076 *	0.055
Spinal Spurs W1	0.104 **	0.092 *
Triglycerides W1	0.069 *	0.006
Ulcer/Stomach Ailment W1	0.129 **	0.059 *
Stress W1	--	0.249 **
Stress W2	0.249 **	--

Note: ** = Correlation is significant at the 0.01 level (2-tailed). * = Correlation is significant at the 0.05 level (2-tailed).

**Table 2:** Correlations between stress at both waves and diseases/biomarkers at Wave 2.

	Stress W1	Stress W2
Arthritis/Rheumatism W2	0.098 **	0.057 *
Cancer W2	0.009	0.057 *
Diabetes W2	0.060 *	0.044
Gout W2	0.092 **	0.055
Heart Disease W2	0.079 **	0.012
High Blood Pressure W2	0.062	0.042
IL-6 W2	0.063 *	0.018
Kidney Disease W2	0.070 *	0.106 **
Liver/Gall Bladder W2	0.049	0.048
Lower Respiratory Disease W2	0.055 *	0.009
Spinal Spurs W2	0.106 **	0.067 *
Stroke W2	0.077 **	−0.029
Triglycerides W2	−0.040	−0.019
Ulcer/Stomach Ailment W2	0.063 *	0.112 **
Urinary Cortisol W2	0.029	0.033

Note: ** = Correlation is significant at the 0.01 level (2-tailed). * = Correlation is significant at the 0.05 level (2-tailed).
